# High-resolution epidemic simulation using within-host infection and contact data

**DOI:** 10.1186/s12889-018-5709-x

**Published:** 2018-07-17

**Authors:** Van Kinh Nguyen, Rafael Mikolajczyk, Esteban Abelardo Hernandez-Vargas

**Affiliations:** 1 Frankfurt Institute for Advanced Studies, Ruth-Moufang-Str. 1, Frankfurt am Main, 60438 Germany; 2 Helmholtz Centre for Infection Research, Inhoffen Str. 7, Braunschweig, 38124 Germany; 3 German Centre for Infection Research, Site Braunschweig-Hannover, Germany; 4 Hannover Medical School, Hannover, Germany; 5 Martin-Luther-University Halle-Wittenberg, Halle (Saale), Germany

**Keywords:** High-resolution, Epidemic, Simulation, Within-host infection, Age-structure, Contact network, Ebola virus

## Abstract

**Background:**

Recent epidemics have entailed global discussions on revamping epidemic control and prevention approaches. A general consensus is that all sources of data should be embraced to improve epidemic preparedness. As a disease transmission is inherently governed by individual-level responses, pathogen dynamics within infected hosts posit high potentials to inform population-level phenomena. We propose a multiscale approach showing that individual dynamics were able to reproduce population-level observations.

**Methods:**

Using experimental data, we formulated mathematical models of pathogen infection dynamics from which we simulated mechanistically its transmission parameters. The models were then embedded in our implementation of an age-specific contact network that allows to express individual differences relevant to the transmission processes. This approach is illustrated with an example of Ebola virus (EBOV).

**Results:**

The results showed that a within-host infection model can reproduce EBOV’s transmission parameters obtained from population data. At the same time, population age-structure, contact distribution and patterns can be expressed using network generating algorithm. This framework opens a vast opportunity to investigate individual roles of factors involved in the epidemic processes. Estimating EBOV’s reproduction number revealed a heterogeneous pattern among age-groups, prompting cautions on estimates unadjusted for contact pattern. Assessments of mass vaccination strategies showed that vaccination conducted in a time window from five months before to one week after the start of an epidemic appeared to strongly reduce epidemic size. Noticeably, compared to a non-intervention scenario, a low critical vaccination coverage of 33% cannot ensure epidemic extinction but could reduce the number of cases by ten to hundred times as well as lessen the case-fatality rate.

**Conclusions:**

Experimental data on the within-host infection have been able to capture upfront key transmission parameters of a pathogen; the applications of this approach will give us more time to prepare for potential epidemics. The population of interest in epidemic assessments could be modelled with an age-specific contact network without exhaustive amount of data. Further assessments and adaptations for different pathogens and scenarios to explore multilevel aspects in infectious diseases epidemics are underway.

**Electronic supplementary material:**

The online version of this article (10.1186/s12889-018-5709-x) contains supplementary material, which is available to authorized users.

## Background

Epidemics of infectious diseases are currently listed among the potential catastrophes that can set the world back in the next decades [1]. Overwhelming research efforts have been developed to predict the danger of the epidemics but their crisis nature often left scientists no better option than learning from the past [1, 2]. Confronting outbreaks of emerging infections, however, requires a swift response and thus the ability to evaluate quickly and early possible outcomes [1]. As such, computer simulations of epidemic models undoubtedly hold the potential as the first-aid toolbox for decision making amid the crisis [1, 3, 4].

Computational approaches in epidemic modelling date back a few centuries ago [5]. Since then, an overwhelming amount of research has been conducted and contributed profoundly to understanding of epidemic control and prevention [5, 6]. The aims of epidemic modelling are to address questions such as whether or not a substantial population fraction is getting infected? how large would the outbreak spread? or how can the outbreak be mitigated with intervention approaches at hand? among others [7, 8]. Answering these questions requires the quantification of these models using disease data on a population level [7, 9–11], which are often delayed [12, 13], under-reported [14], or not readily available [9]. As a result, epidemic models using population data, while progress understanding on diseases, might have limited applications to an ongoing epidemic [15]. A potentially useful way to predict future disease dynamics is using within-host processes [11].

**Fig. 1:**
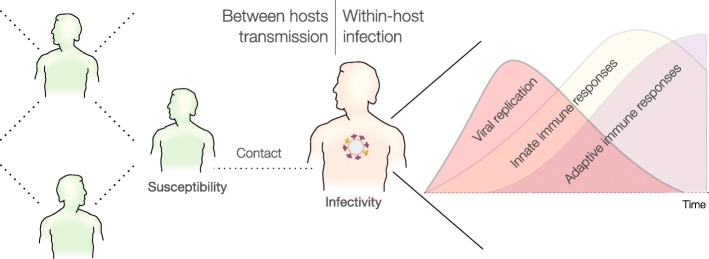
Schematic presentation of the within-host infections processes and their relation to transmission parameters. At the within-host level, viral replication and immune responses race with each other that eventually determines an individual infectivity, for example, his symptoms and possibly behaviours. At the between hosts level, infected individuals make contact(s) with susceptible individual(s) that eventually lead to a transmission, depending on both the infectivity of the infectious and the susceptibility of the susceptible. Noting that while the contact network can be fixed, the portion actively partake in epidemic spreading dynamically change over time [75]

In reality, the within-host infection process determines key parameters in a disease transmission ([16–18], Fig. 1). In an infected subject, interactions between the pathogen and immune responses shape the pathogen dynamics which, ultimately, define the incubation period, the transmission potential, and the recovery rate [11, 18]. It is also evident that susceptibility to infection is not the same for all the susceptible but, among others, it is highly correlated with a subject’s age due to age-related changes of the immune system [19, 20]. These observations together point towards some inherent biological processes where individual dynamics can help to predict population-level epidemics. Differences in the within-host infection profile as well as the susceptibility to infection complicate greatly epidemic models but at the same time underline their influential roles in determining epidemics features and intervention effects [21–23].

The interplays between within-host infection and between hosts transmission led to arising attempts connecting the two levels [4, 17, 18, 24–28], but the approach is still at its infancy [16]. On one hand, most of these models were conceptual and theoretical [16, 28] or relied on assumptive and previously obtained parameter estimates [10, 11, 29]. We propose that this limitation can be overcomed by using explicitly within-host infection model as a unit in epidemic simulations. This allows not only a high level of heterogeneity to be expressed, but also the study of stochasticity effects in epidemic spreading.

On the other hand, implementations of population level models were either a general representation using probabilistic assumptions [30, 31] or a computationally demanding implementation of a particular population [11, 29, 32]. These approaches, while able to recover valuable insights, may not be representative and accessible for another population of interest, because either none or large amount of data were needed. In this case, an implementation of an epidemic model with social mixing attributes, i.e., number of contacts per day and to whom the contact occurs, could be less computationaly intensive [33], consistent across populations [34, 35], and more representative for a realistic disease spreading process [36, 37].

Improving understanding on EBOV transmission is crucial: EBOV can cause a large scale outbreak with a high fatality rate [14] and it re-emerges continuously [38]; as of May 2018, a total of 32 EBOV disease cases have been reported from Democratic Republic of the Congo, including 18 deaths [38]. Based on our previous studies of the within-host EBOV infection [39–41], we brought forward a within-host infection model to study the transmission fitness of EBOV at the population level. We built a network model based on social contact data [34] and the respective epidemic simulation algorithm that embedded the within-host model into the contact network. Thus, this multiscale model was data-informed at each level of the epidemic process.

Our multi-scale model aimed to express the link between pathogen load and transmission potential of an infected host, the immune responses to the infection and vaccination, and the heterogeneity of the contact frequency distribution in a population. The parameters obtained from model simulations were compared to those estimated from population-level data and empirical observations. The results showed that within-host infection model reproduced estimates of the transmission parameters and allowed detailed evaluations of the effects of intervention timing on the course of the epidemic. Implementations of the network model from social contact data was straightforward and scalable for large simulations on high-performance computer clusters. In that capacity, epidemic assessments and preparations can be conducted quickly, ahead of time, and with high-resolution requirements.

## Methods

In an EBOV-infected subject, the immune system components dynamically evolve in response to the virus replication dynamic [42]. As a result, a series of events is triggered determining the infection outcomes such as infectious status, symptoms, recovery, or death [42–44]. In this paper, the EBOV replication dynamic within a host was used to infer its transmission parameters.

### Within-host model

Using virus dynamics and the immune response data within a host, mathematical relations can be defined to test hypothesized infection mechanisms [39, 45]. In this context, non-human primates (NHPs) are the standard animal model for developing EBOV’s therapeutics and vaccines in humans [46–48]. Epidemiological and pharmacological studies reported that a viral load level higher than 10^6^ copies/mL [47, 49] was associated with a higher mortality rate, whereas observations based on experimental data in NHPs showed that a viral load level higher than 10^6^ TCID_50_ was fatal [43, 44]. Here the viral load dynamics were simulated based on a model as follows [41]:

1 $$ \frac{dV}{dt} = r_{V} V\left(1-\frac{V}{K_{V}}\right) \left(\frac{V}{I_{n}+V}\right) \left(1 - \frac{{Ab}}{K_{Ab}}\right)  $$

where *r*_*V*_,*K*_*V*_, and *I*_*n*_ denote the viral replication rate, the carrying capacity of the infected host, and a threshold expressing a lag-phase of viral replication. In particular, a logistic growth was assumed for EBOV, with a short delay when the virus level was low. The parameter *K*_*Ab*_ represents the strength of the immune system at which the antibody titre (*Ab*) completely inhibits the viral net growth rate [41, 44], i.e., it was assumed that the higher antibody level required to inhibit the viral replication the weaker the immune strength. An antibody titre level of 10^4^ appeared to be protective in NHPs and it required approximately one week after vaccination to reach this threshold [44]. The model parameters were obtained previously [41] using two experimental datasets on NHPs [43, 44]. The antibody dynamic (*Ab*) was also fitted in [41] to the data of NHPs vaccinated with a recombinant vesicular stomatitis virus vaccine (rVSV-EBOV) [44]. This vaccine had shown a high efficacy in human [48]. Details of model fitting, data, and the parameter set can be found in [41] and “Availability of data and materials” section.

### Simulated subject-specific infection course

To simulate subject-specific infection course, the antibody response strength *K*_*Ab*_ was varied from a normal level of approximately 10^2.5^ [44, 50] to the highest observed level of 10^4.5^ [44]. This parameter was assumed to follow a U-shaped function of an individual’s age, where infants and elderly have a higher susceptibility ([19], Fig. 1) (the extracted function is presented in “Availability of data and materials” section). As the infective dose can alter the course of infection [51], the initial condition *V*(0) of the model Eq. 1 was varied depending on from whom a subject acquired the infection, i.e., the infection dose was assumed as equal to the lethal dose (*V*_*c*_=10^0.15^ [41]) times the transmission potential of a subject transmitting the infection. Here we assumed a direct relation between the transmission potential and the viral load at the time of infection [16], i.e., the transmission potential *p*_Trans_(*t*)=*V*(*t*)/*K*_*V*_. Note that *p*_Trans_(*t*)=1 does not guarantee a successful transmission, but it was considered collectively with its contact’s susceptibility and with the existence of such a contact’s (details in “Availability of data and materials” section).

### Infection outcomes definitions

**Fig. 2:**
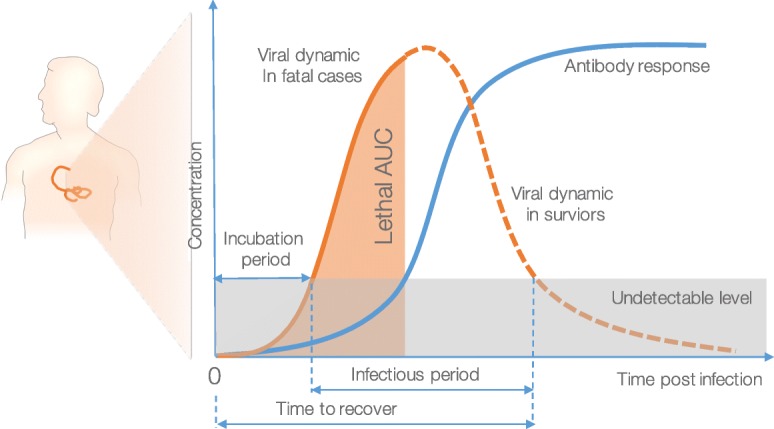
Simulated infection course using within-host infection dynamics. The viral replication, the antibody dynamics, and their interaction were modelled to define epidemiological parameters. It is assumed that when the EBOV-specific antibody concentration reaches a certain threshold, it can inhibit the viral replication [76]. The total viral load under the curve (AUC) in lethal cases is used to define infection outcomes [77]

Empirical observations from EBOV infected human and NHPs showed that the time from symptom onset to death was approximately one week [43, 44, 52]. Based on this, we used the total area under the viral load curve (AUC) seven days post-infection obtained from the subjects that died as a threshold above which the infection is lethal, i.e., $\text {AUC}_{7} = \int _{0}^{7} V(t)dt$. Otherwise, infected subjects were assumed to have recovered once the viral load was no longer detectable (Fig. 2). Depending on the infective dose and the adaptive immune response strength, the infection model manifests different viral dynamics and consequently the infection outcomes. Based on that, we defined the transmission parameters as in Table 1A-C (detailed implementations can be seen in “Availability of data and materials” section).

**Table 1:** Definitions of transmission parameters

	Measure	Definition
A	Incubation period	the interval between exposure to a pathogen and initial occurrence of symptoms [79] was defined as from the infection time to the first time the viral load crosses over the detectable threshold (Fig. 2).
B	Time from symptom onset to recovery [79]	defined as the interval between the first day of detectable viral load and the first day the viral load becomes undetectable (Fig. 2).
C	Time from symptom onset to death [79]	defined as the interval between the first day with detectable viral load and the day the area under the viral load curve (AUC) crosses the reference threshold *AUC*_7_ (Fig. 2).
D	Basic reproductive number (R0)	calculated based on the network of infected subjects at the end of an epidemic in an initially susceptible population. In a network model, this equals the mean degree distribution of the infected network, considering a directed network without loops (e.g., Fig. 5). The R0 by age-group was also calculated in the same fashion based on the assigned age-attribute. In epidemics with intervention or when the population is not fully susceptible, the R0 is called the *effective reproduction number* (Re).
E	Final infected fraction	the proportion of infected nodes at the end of an epidemic simulation.
F	Case-fatality rate	the proportion of nodes who died as a result of EBOV infection counted at the end of an epidemic simulation.

### Network model

The European’s contact patterns survey data [34] were used to generate a network model reflecting the number of contacts and the mixing patterns among age-groups. This dataset is currently the largest collection available on human contact patterns; moreover, a similar pattern has also been observed outside Europe [35]. To make the contact pattern more specific to an EBOV-affected population, the age distribution of Freetown city in Sierra Leon [53] was used to weight the contact pattern towards this area. In particular, while the contact frequency and pattern remain the same, these data were imposed on a network that has more young and less elderly subjects. A detailed description of the network implementation can be found in Availability of data and materials.

Because EBOV spreads through direct contacts with infectious subjects [51], and the highest risk of infection is contacting with blood, faeces, and vomit [54], we used only the data of physical contacts and excluded those contacts with a duration of less than five minutes. In the 2014 EBOV epidemic, an important transmission route was through contacting with the deceased who had not been buried [2]. To account for this we considered deceased EBOV-infected subjects as infectious until they were buried. During the 2015 EBOV epidemic, the time from death to burial was from one to two days on average, but it can be a week [55]. This information was used to formulate a truncated normal distribution for the time from death to burial, i.e., the distribution was truncated at zero and seven and had unit mean and variance (detailed implementations can be seen in Availability of data and materials).

### Transmission outcomes definitions

**Fig. 3:**
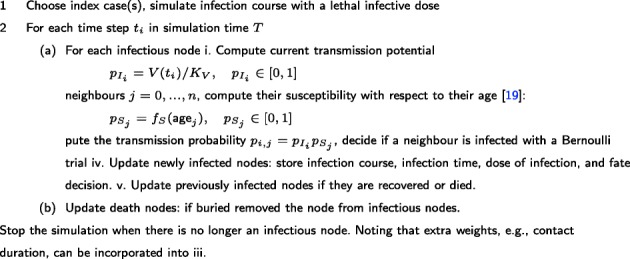
EBOV epidemic simulation process

To obtain EBOV’s epidemic metrics, the within-host infection model was embedded into the network model. Simulations of EBOV epidemic are detailed in Fig. 3. In short, a network of ten thousand nodes was generated. Scenarios in which the population was randomly vaccinated during one-week vaccination program were tested and compared to a control simulation without vaccination. To isolate the effect of vaccination, we assumed that no other interventions were in place, e.g., no treatments were provided and no quarantine or isolation programs occurred. For each scenario, one thousand simulations were performed, each of which started with a single random index case. Each time when a contact occurred, the viral load at that time point was extracted to determine the transmission potential. Next, the susceptibility of the contact persons was computed as a function of their age [19]. A Bernoulli trial was then used to determine if the contact results in an infection given the overall transmission probability. If the transmission succeeded, for the newly infected subject his/her own infection profile was computed. Based on simulation outputs, the epidemic outcomes were determined as in Table 1D-F (detailed implementations can be seen in Availability of data and materials).

### Potential weaknesses and remedial approaches

The following assumptions were used given the lack of specific experimental data, but further efforts to produce the data can be done to address the issues listed here:

(i) Secondary antibody responses were assumed to be similar to primary responses. This underestimates the effect of vaccination strategies conducted before an epidemic. Experimental studies on secondary immune responses to EBOV infection are needed, especially those with a longer follow-up period.
(ii) The transmission potential was assumed as directly related to viral load. While this is reasonable, non-linear relationships might exist [16, 28]. Dedicated animal experiments to define the exact relationship between the viral load and the ability to transmit the virus are needed.
(iii) The contact pattern was assumed similar for Ebola affected regions as in European countries. Although the contact patterns seemed similar across countries [34], a more sociable population would have higher contact rate and thus increase R0. As collecting this data for all countries can be laborious, simulation studies addressing the effect of contact patterns on the connectivity in network models are needed.
(iv) Infection status was assumed to have no influence on the network structure, except that those buried were removed from the network. This could overestimate R0 [56]. Taking people’s behaviour changes into epidemic modelling remains a grand challenge [56].
(v) Susceptibility to EBOV infection was assumed similar to a general viral infection disease. Studies on susceptibility functions are lacking and require more attention of the infection research community.

### Computational implementation

The simulations were written in vectorized R language [57]. Ordinary differential equations (ODEs) were solved with deSolve package [58]. The network was generated as an adjacency matrix and was visualized with the package igraph [59]. Computations of infection dynamics of the newly infected nodes were done during the epidemic simulation after obtaining its infective dose and immunization status. For nodes with identical conditions, their infection courses were copied instead of recomputing the ODEs for speed (Availability of data and materials). Repeated runs of epidemic simulations to obtain uncertainty estimates were done on computer clusters of the Center for Scientific Computing (CSC) of the Goethe University Frankfurt. Distribution of computation resources was sent from within R to SLURM Workload Manager.

## Results

### Basic transmission characteristics

Table 2 shows that the within-host infection model captured well the population-level transmission parameters. The results suggest that in contrary to using outbreak data, employing within-host infection model can provide this information prior to outbreaks, and even for a scenario where a pathogen *X* has never caused an epidemic before [60].

**Table 2:** Simulated population-level transmission parameters based on within-host infection model

	Range and medians (in days)	
Parameter	Simulated	Literature
Incubation period	2.6–12.4 (3.8)	3.35–12.7 (7) [79]
Time from symptom onset to recovery	6.9–17.6 (9.7)	2–26 (10) [79]
Time from symptom onset to death	8.1–15.1 (9)	3–21 (9–10) [79]

### The network model

**Fig. 4:**
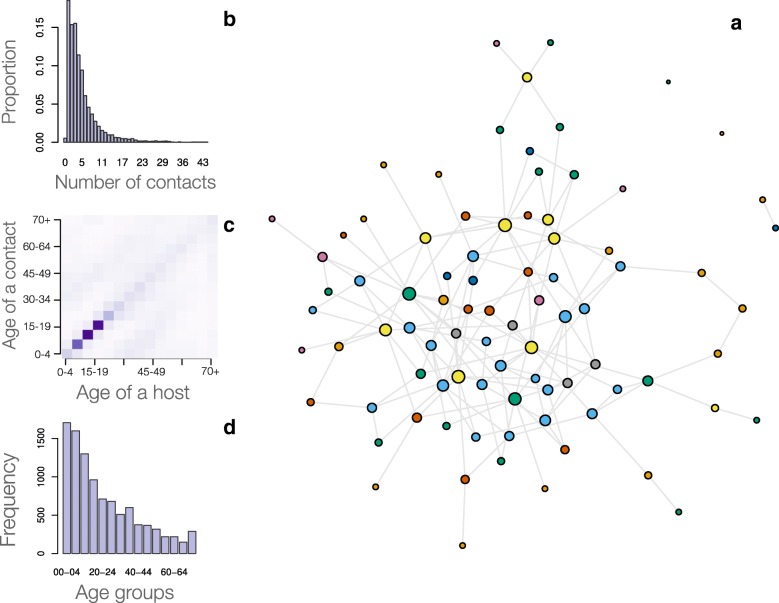
Required data and an example of an age-structure network model. (**a**) Generated network of one hundred individuals that mimics distribution of physical contact, contact matrix, and population age structure. The node’s size reflects its number of contacts. Nodes in the same age-group have the same colour; (**b**) Distribution of number of physical contacts shows a majority of individuals have a few physical contact per day [34]; (**c**) A heat map of contact matrix shows higher contact frequencies in darker shades. The matrix reflects the assortative pattern of human contacts, that is people contact mainly with their peers, follow by their children or parents. The age-group with the highest contacts are teenagers and young adults [34]; (**d**) Reconstructed age-structure of Sierra Leon population based on Statistics Sierra Leone and ICF International data [53]

Figure 4 shows an example of the generated networks and its required data. In particular, given a network of size $N \in \mathbb {N}$, each node was assigned an age such that the network’s age-distribution resembled that of the target population. Subsequently, nodes were assigned a number of contacts per day following a defined contact distribution of interest. Finally, the algorithm visited each node to generate the defined number of contacts, not at random but following a defined contact matrix. The network was returned as an adjacent matrix that is compatible to available network analyses algorithms, e.g., igraph, graph-tool [61, 62]. Storing data as a sparse matrix, a regular installation of R could generate reliably networks of 10-20 thousand nodes with the generation time 6-10 minutes on a single thread Intel Core i7, 8GB RAM. Note that R theoretically can only handle a maximum square matrix ≈44721 rows and columns.

### Calculating basic reproductive number (R0)

**Fig. 5:**
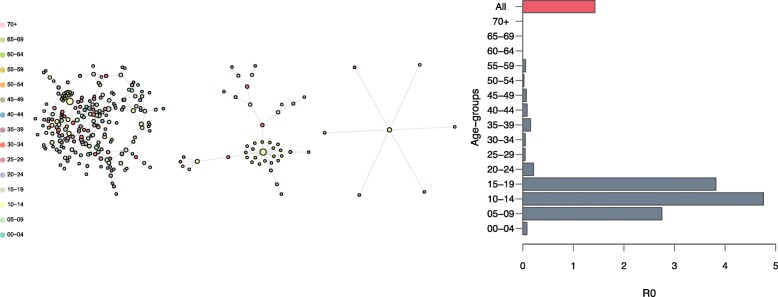
Left. Three examples of infected networks. The three networks were randomly chosen from the simulated epidemics. Uninfected nodes were removed and the network is plotted. Based on these, the R0 was calculated based on the edges assuming a directed network, i.e., each edge counted in only one direction. Right. Estimates of the basic reproductive number without any intervention, overall and by age-groups. Simulations of a network of size ten thousand during a period of one year. One thousand simulations were run, each time with a random index case. At the end of each simulation, networks of infected nodes were extracted to compute the average number of secondary infections

After each simulation, the uninfected nodes were removed from the initial network. Then the basic reproduction number was calculated as the average network degree, considering the network as a directed and without loop network (Fig. 5-Left). Simulation results showed that the overall estimate of R0 was 1.43 (Fig. 5-Right). However, the estimates differed across age-groups with the highest of 4.7 for the group of 10-14 years of age. Intuitively, the age-groups with a higher contact rate had also a higher R0. Simulations of epidemics with varied intervention strategies showed that Re can be reduced below unity if a vaccination program with 85% coverage was deployed at the time as far as five months before the introduction of the index case (time zero) or as late as one week after that (Additional file 1: Figure S1). This coverage threshold was tested as it is the highest vaccine coverage currently achieved worldwide for some diseases, e.g., Hepatitis B, measles, and polio [63]. Late initiations of a similar intervention from one to five months after the time zero gradually shifted the Re to the outbreak domain.

A lower vaccination coverage of 33%, while substantially reduced the epidemic size, still posed a potential of a large outbreak regardless the timing of vaccination program (Additional file 1: Figure S1). This coverage was tested as it is a theoretically protective threshold in stochastic and heterogeneous mixing models, i.e., 1-1/R0 [7, 64]. Note that the tested time window of five months before the appearance of the index case was chosen based on the windows of opportunity for EBOV vaccination [41]. As of now, no detailed data are available on the secondary antibody responses to EBOV; it was therefore assumed that secondary responses are similar to the primary responses.

### Case-fatality rate

Simulations showed that the case-fatality rate in the absence of intervention was about 91% (Additional file 2: Figure S2), which falls within the range of literature estimates of 0.4 to 0.91 [79]. Note that the simulations assumed worst case scenarios where no other inverventions were done except the vaccination. Furthermore, simulation results showed that all the intervention strategies mentioned previously reduced the case-fatality rate. These results highlighted a benefit of vaccination programs even if they were late: reducing the disease severity in newly infected subjects after the introduction of the vaccination program. As such, relying on R0 as the solely indicator for evaluating intervention programs would overlook this life-saving aspect.

### Epidemic final size

**Fig. 6:**
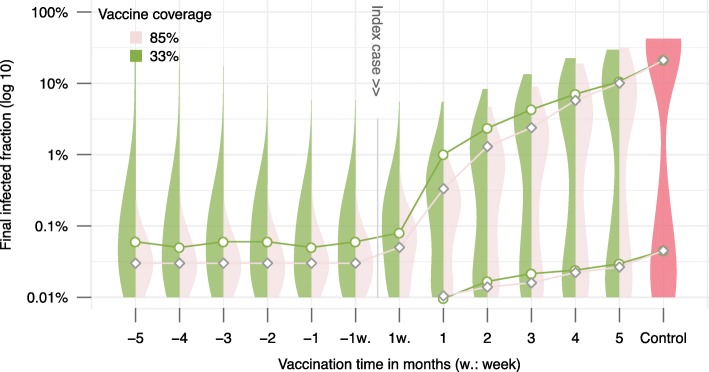
Distribution of the final infected fraction in different timing and coverage of vaccination strategies. A synthetic population of ten thousand individuals was generated. One thousand simulations were run to simulate the epidemic in the time course of one year. Each time, one individual was chosen randomly as the index case. Circles, diamonds, and connected lines are median. Filled areas are the corresponding non-parametric densities estimates [78]. Two median values are presented for multi-modal density estimates, determining by inflection points

Theoretical analyses of stochastic epidemic models showed that when R0 is larger than unity, the final size of an epidemic converges to a bimodal distribution: either the epidemic dies out with a small number of infected cases or the epidemic takes off to a normal distribution with a high number of cases [7]. Our simulation results recreated this epidemic behavior (Fig. 6). Without intervention, EBOV had approximately 50% probability to infect more than half the population. The introduction of vaccination programs at the two previously mentioned coverages and at any vaccination time points under assessment scaled down the epidemic size (Fig. 6). The earlier the vaccination programs were deployed, the closer the epidemics size distribution resemble to a uni-modal distribution centered at a low infected fraction. The high vaccine coverage strategy effectively eliminated the possibility of having a major outbreak infecting a large proportion of the population. This was achieved when the vaccination programs were deployed at any time point from one week to five months before time zero.

Figure 6 also shows that a random vaccination program covering 33% of the population one week before the time zero reduced the final size by more than 100 times compared to a no intervention scenario. However, the low coverage strategy still showed a small probability that the epidemic becomes major, whereas the high coverage strategies did not. Vaccination programs deployed during the epidemics also substantially reduced the epidemic’s size: the vaccination program conducted one month after time zero still reduced the final size by more than ten times. Furthermore, these interventions were not only able to reduce the final size, but they could also increase the epidemics extinction probability (Fig. 6).

## Discussion

In estimating parameters and designing prevention strategies for infectious diseases, it is important to take into account mechanisms underlying the heterogeneity in immune responses [65] and in the population [34]. This paper combined a within-host immunological model of Ebola virus (EBOV) infection and an age- and demographic-specfic contact network to study EBOV epidemic. The multi-scale model reproduced major characteristics of EBOV epidemics and allowed finer assessments of the timing of vaccination strategies.

Estimates of the EBOV’s incubation period suggested a contact tracing period of three weeks for Ebola epidemics, matching the current WHO’s recommendation of 21 days [66]. Estimates of the delay distributions agreed with information that EBOV infected subjects can be infectious from day 3 up to three weeks post infection [79]. Understanding of these delay distributions is critical in clinical and epidemiological perspectives [67]. These distributions, however, are often only partially observed in practice: it is difficult to know the exact time of exposure to the pathogen or to have complete outbreak data [68, 69]. As such, parameter estimation of these distributions have been relied on testing and comparing distributional assumptions [69]. The mechanistically generated transmission characteristics using virus dynamics remarkably resemble literature estimates. This suggests some inherent biological processes where individual dynamics accumulate to produce population-level epidemics. This approach is thus promising and practical given the accumulating experimental data on varieties of pathogens, such as a disease *X* [60] that as yet unknown in epidemic contexts.

To determine infection outcomes, the threshold AUC_7_ was chosen based on suggestions derived from empirical data in humans [52] and non-human primates [43, 44]. Simulations of the epidemics using this threshold reproduced estimate of EBOV case-fatality rate (Additional file 2: Figure S2), suggesting that the use of total viral load (AUC) as a criterion for determining infection outcomes is appropriate. It is worth noting that the calculated case fatality rate was based on the assumption of worst case scenarios where no treatments were provided. In practice there were medical approaches to reduce disease severity. Scientific literature has shown that for severe cases of EBOV infection (equivalent to unvaccinated subjects or those vaccinated too late in our model), supportive treatments increased substantially the survival chance [70]. Although a more precise threshold criterion is desirable, it might not be feasible to obtain it in practice considering the inherent ethical reasons. Thus a similar criterion as used here could be considered when adapting this approach to other diseases, but ideally derived from dedicated experimental data.

Different classes of network models have been proposed, but they cannot reproduce properties observed in real world networks [71]. In addition, choices of theoretical network structure used for simulation can alter epidemic outcomes [30]. Thus, a network model built from empirical data would provide a more solid ground for epidemic simulations. Apart from mimicking the contact data properties, our network model can express age-related infection traits via the assigned age attribute. It was used in this paper to express individual differences in the susceptibility and immune response to viral infection—the crucial elements in a realistic disease transmission. Although contact data might not be available for a certain target area, the assortative patterns of human contacts and the highly skewed distribution of the number of contacts might hold true across regions [34, 35]. Thus, this paper presents a simple way to bring empirical contact data into epidemic modelling studies.

Our network model currently can only simulate epidemics in a population of size 10–20 thousand. This is because of the limit in R with the theoretical maximum square matrix size of approximately 45000 [57]. A more efficient storing of the network could extend the network size, such as a lazy evaluation used in igraph [61]. However, it could be more realistic to have several communities amount to a large population size instead of a large single network. This can be implemented by generating different communities across computers and allow them to communicate, speeding also the computation processes [72]. In this case, additional data are needed to model the communication among the communities, such as transportation network and immigration flow.

Regarding EBOV epidemics, previous R0 estimates based on epidemic data varied strongly, depending on model choices and assumptions [79]. Our estimate of R0 was 1.4 which is within the range of the previous estimates, ranging from 1.2 to 2.6, with some exceptional estimates up to 4.7 and 8.3 [79]. Notably, we showed that the estimates differed by age-groups with the highest of 4.7 for the group of 10–14-years of age. Although these estimates depend on Sierra Leon’s age-structure, the differences of R0 estimate stress the role of the age-structure and contact patterns in the estimation of R0, prompting that age-specific intervention strategies should be considered. The estimates by sub-groups also single out the effort required to control the epidemic [73]. With the assumptions used in our models, targeting interventions to the group 5–20-years of age would be the most effective strategy. Note that the differences of R0 by age-group also provide us an explanation of the wide variation of the previous estimates of R0 where different samples were used [79]. As the R0 estimates were larger in sub-groups, our results confirmed that the critical vaccine coverage also needed to be larger to ensure eradication of the epidemic [64, 74]. Using our approach, we have shown further that while a low coverage could not completely eradicate the epidemic, it could largely reduce both the size and severity of an epidemic—which is worth pursuing in cases of lacking resources to reach an optimal threshold.

## Conclusion

Throughout this paper, we showed the possibilities to investigate practical and intriguing questions using a within-host viral dynamic model and an age-structured network model. The advantages of using explicitly within-host dynamics are the availability of experimental data, the possibility of conducting experiments to characterize transmission parameters, and the ability to provide high-resolution subject-specific responses to infection. The advantages of using an age-structured network model are its simple implementation, its representativeness for disease transmission, and the availability of the age-structured data. Therefore, immunological studies of infectious agents could be seamlessly integrated into studies of between hosts transmission, promoting evidence-based public health practices.

## Additional files

Additional file 1 **Figure S1.** Estimates of the reproductive number in different vaccination schemes. Simulations of a network of size ten thousand during a period of one year are performed. One thousand simulations were run, each time with a random index case. At the end of each simulation, the network of infected nodes was extracted to compute the average number of secondary infections. (PDF 27 kb)

Additional file 2 **Figure S2.** Case-fatality rate in different vaccination schemes. Simulations of a network of size ten thousand during a period of one year are performed. One thousand simulations were run, each time with a random index case. At the end of each simulation, the network of infected nodes was extracted to compute the case-fatality rate. (PDF 28 kb)

## Abbreviations

AUC: Area under the curve
EBOV: Ebola virus
NHPs: Non-human primates
R0: Basic reproduction number
Re: Effective reproduction number
VSV: Vesicular stomatitis virus
WHO: World health organization
