# Supplementary figures and images for: High-resolution epidemic simulation using within-host infection and contact data
Source: BMC Public Health. 2018 Jul 17;18:886. doi: 10.1186/s12889-018-5709-x (PMC6050668; doi:10.1186/s12889-018-5709-x)

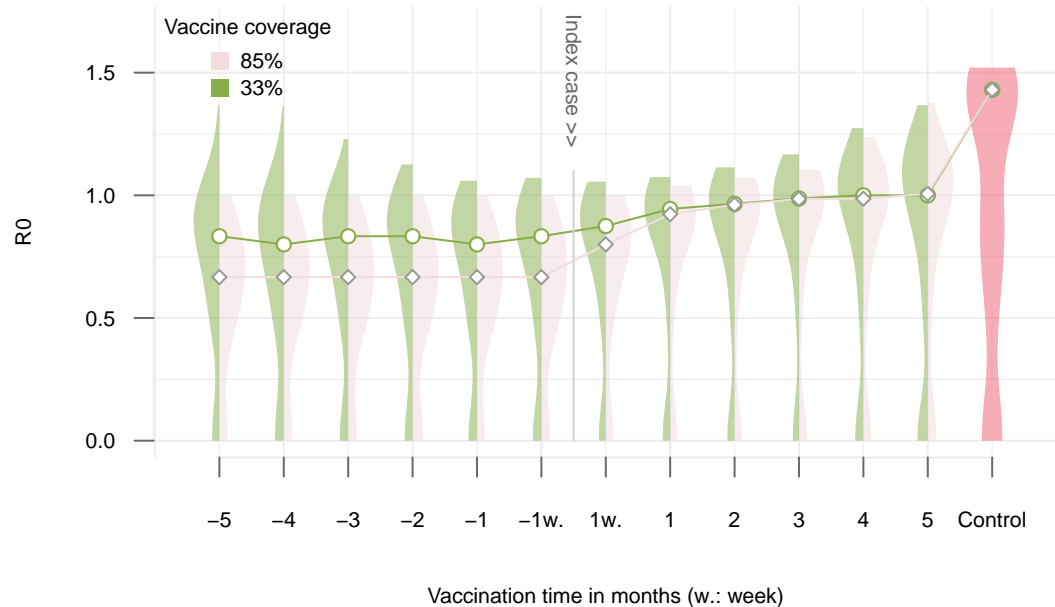

Supplement: Supplementary file 1 — Figure S1. Estimates of the reproductive number in different vaccination schemes. Simulations of a network of size ten thousand during a period of one year are performed. One thousand simulations were run, each time with a random index case. At the end of each simulation, the network of infected nodes was extracted to compute the average number of secondary infections. (PDF 27 kb) [file 12889_2018_5709_MOESM1_ESM.pdf]

Case-fatality rate

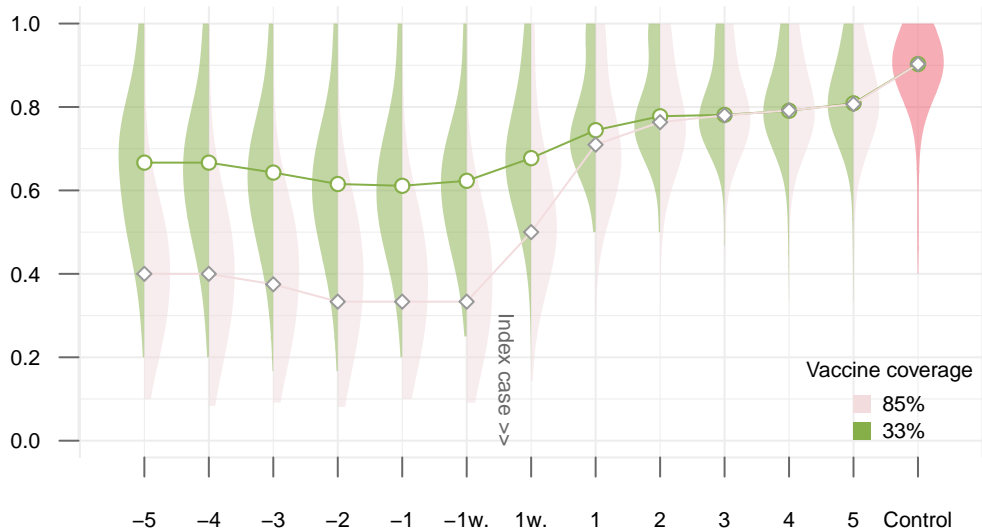

Vaccination time in months (w.: week)

Supplement: Supplementary file 2 — Figure S2. Case-fatality rate in different vaccination schemes. Simulations of a network of size ten thousand during a period of one year are performed. One thousand simulations were run, each time with a random index case. At the end of each simulation, the network of infected nodes was extracted to compute the case-fatality rate. (PDF 28 kb) [file 12889_2018_5709_MOESM2_ESM.pdf]
